# Synthesis of two novel bio-based hydrogels using sodium alginate and chitosan and their proficiency in physical immobilization of enzymes

**DOI:** 10.1038/s41598-022-06013-0

**Published:** 2022-02-08

**Authors:** Fateh Shakeri, Shohreh Ariaeenejad, Marzieh Ghollasi, Elaheh Motamedi

**Affiliations:** 1 Department of Cell and Molecular Biology, Faculty of Biological Sciences, Kharazmi University, Tehran, Iran; 2 Department of Systems and Synthetic Biology, Agricultural Biotechnology Research Institute of Iran (ABRII), Agricultural Research Education and Extension Organization (AREEO), Karaj, Iran; 3 Department of Nanotechnology, Agricultural Biotechnology Research Institute of Iran (ABRII), Agricultural Research Education and Extension Organization (AREEO), Karaj, Iran

**Keywords:** Biochemistry, Chemistry, Materials science, Nanoscience and technology

## Abstract

Herein, four novel and bio-based hydrogel samples using sodium alginate (SA) and chitosan (CH) grafted with acrylamide (AAm) and glycidyl methacrylate (GMA) and their reinforced nanocomposites with graphene oxide (GO) were synthesized and coded as SA-g-(AAm-co-GMA), CH-g-(AAm-co-GMA), GO/SA-g-(AAm-co-GMA), and GO/CH-g-(AAm-co-GMA), respectively. The morphology, net charge, and water absorption capacity of samples were entirely changed by switching the biopolymer from SA to CH and adding a nano-filler. The proficiencies of hydrogels were compared in the immobilization of a model metagenomic-derived xylanase (PersiXyn9). The best performance was observed for GO/SA-g-poly(AAm-co-GMA) sample indicating better stabilizing electrostatic attractions between PersiXyn9 and reinforced SA-based hydrogel. Compared to the free enzyme, the immobilized PersiXyn9 on reinforced SA-based hydrogel showed a 110.1% increase in the released reducing sugar and almost double relative activity after 180 min storage. While immobilized enzyme on SA-based hydrogel displayed 58.7% activity after twelve reuse cycles, the enzyme on CH-based carrier just retained 8.5% activity after similar runs.

## Introduction

Boosting the catalytic activity and operational stability of enzymes and facilitating their recovery and reuse, are considered the primary objectives of enzyme immobilization^1,2^. To take advantage of practical utilization of the immobilized enzymes, some contributing factors should be noticed, such as choosing the proper immobilization protocols and the appropriate support materials for minimizing the risks of subunit dissociation, deactivation, aggregation, autolysis, or leaching of the enzymes^3–5^. Among numerous potential supports, bio-based hydrogels are exceptionally attractive options for enzyme immobilization because they not only can meet the requirements of the enzymes but also can withstand conditions of the enzymatic reactions^6–8^. Hydrogels are 3D cross-linked porous polymeric networks containing various hydrophilic groups with a huge capacity to absorb and conserve water^9,10^. These carriers could be produced under mild processes using abundant, green, and relatively low-priced natural polymers (i.e., chitosan, starch, cellulose, alginate, pectin)^11–13^. Moreover, such hydrogels as smart biomaterials have good biocompatibility and can provide a suitable microenvironment for biomolecules^11^. Among all natural polysaccharides studied for the synthesis of hydrogels, alginate and chitosan have been extensively investigated to prepare carriers with various applications in drug delivery, tissue engineering, and enzyme immobilization^14–18^. Chitosan (CH) is a cationic and hydrophilic bio-polymer typically obtained from alkaline hydrolysis of chitin (an amino polysaccharide extracted from the exoskeleton of crustaceans)^19–21^. Sodium alginate (SA) as anionic polysaccharide is derived from alginic acid which is obtained using alkaline treatment of brown marine algae^22–24^.

Typical studies on the applications of the hydrogels in enzyme immobilization have used covalent attachment of an enzyme with a functionalized hydrogel. However, our previous reports confirmed that the robust physical enzyme immobilization on hydrogel carriers would be provided using the synthesis of a modified hydrogel that was capable to interact proficiently with enzymes via electrostatic interactions, hydrogen bonds, or Van der Waal's forces^25–28^. In such physical immobilization cases, the isoelectric point (PI) of the enzyme played a key role in the conjugation process. In fact, the as-prepared hydrogels were simply swelled in the buffer solutions containing enzymes at pHs below the PI^25^. At this condition, the enzyme has a positive charge, and so if the hydrogel had a negative charge at this pH, it would result in the preparation of stable enzyme-carrier conjugates through effective electrostatic attractions^26^.

Considering all these backgrounds, we continued our research by reinforcing hydrogels with graphene oxide nano-filler^29^ to synthesize dual-crosslinked (DC) hydrogel networks and investigating their applications in cellulase enzyme immobilization^27^. Then, we scrutinized the stabilizing mechanism of two immobilized xylanases on the synthesized hydrogels using computational and experimental perspectives^28^. Following our recent studies, herein, we aimed to evaluate the effects of bio-polymer’s charges on the physical immobilization of enzymes onto the hydrogels. To this goal, two different natural polymers, SA and CH have been selected to form the backbone of the hydrogel supports. In our previous works, we utilized acrylic acid (AA) and 2-Acrylamido-2-methylpropanesulfonic acid (AMPS) along with acrylamide (AAm) as grafting monomers on the backbone of the carboxymethyl cellulose (CMC). Nevertheless, ionization of the carboxylate and sulfonate groups of the AA and AMPS on the grafted polymer would lead to a net negative charge of the hydrogels when they swelled in pH ranges above 4.0, and it covered the effect of the biopolymer charges. Hence, in this study, we removed AA and AMPS and used acrylamide and glycidyl methacrylate (GMA) as vinyl monomers to graft SA and CH. Consequently, two different SA- and CH-based pristine hydrogels have been synthesized and marked as SA-g-poly(AAm-co-GMA) and CH-g-poly(AAm-co-GMA), respectively. Moreover, to evaluate the effects of nano-filler on the hydrogel’s structures and performances, two samples reinforced with GO nano-sheets were also prepared and named GO/SA-g-poly(AAm-co-GMA) and GO/CH-g-poly(AAm-co-GMA). Then, the proficiencies of these four hydrogel samples were compared and contrasted in the immobilization of a model recombinant metagenomic-derived xylanase (PersiXyn9). The structural characterization results confirmed that the microstructure, net charge, and water absorption capacity of hydrogel samples were entirely changed by switching the biopolymer backbone from SA to CH and adding GO nano-filler. Moreover, immobilization of PersiXyn9 on these four hydrogel supports resulted in an increase in its thermal and storage stability, improved its catalytic activity and reusability. In general, the best performance in enzyme immobilization has been observed for GO/SA-g-poly(AAm-co-GMA) sample indicating better stabilizing interactions between PersiXyn9 and SA-based hydrogel r than CH-based samples. These findings may broaden the knowledge about relationships between the compositions of the hydrogels and their effects on the immobilized enzymes, which could be valuable in the design and development of novel and effective bio-based hydrogels for the successful engineering of optimal immobilized enzymes.

## Materials and methods

### Materials

Sodium alginate (SA), chitosan (CH), acrylamide (AAm), *N*,*N*′‐methylenebis(acrylamide) (MBA), glycidyl methacrylate (GMA), and ammonium persulfate (APS) were purchased from Sigma-Aldrich, and used for the synthesis of the hydrogel. Natural flake graphite was obtained from Qingdao Dingding Graphite products; H_2_SO_4_ (98%), H_2_O_2_ (30%), KMnO_4_ (98%), were also obtained from Sigma-Aldrich, and utilized for the preparation of graphene oxide (GO). For preparation/isolation and characterization of enzyme, all chemicals including 3,5-Dinitrosalicylic acid (DNS), Bovine serum albumin (BSA), beechwood xylan were from Sigma-Aldrich. The PersiXyn9 was cloned, expressed, purified and characterized according to the previous study in Agricultural Biotechnology Research Institute of Iran (ABRII). The phosphate buffer with appropriate pH and deionized water were used to prepare the samples.

### Synthesis of SA-based hydrogels

Nano-filler (GO) was synthesized according to our earlier works using modified Hummer’s method^27^. Next, SA-based hydrogel samples were prepared by grafting acrylic monomers (AAm and GMA) onto the sodium alginate backbone via free-radical polymerization, in the presence of a chemical cross-linker (MBA). Firstly, SA (500 mg) were weighed and dissolved in deionized water (5 mL). Then, AAm (250 mg), and certain amounts of GMA and MBA were added to the reaction flask, and the mixture was stirred for 15 min. Afterward, 20 mg of initiator (APS) was added and the temperature of the reaction was raised to 80 °C, with continuous stirring. The resultant gel product was synthesized within 5 min, and then it was immersed in copious deionized water for 24 h. Lastly, the swelled hydrogels were filtered to remove excess water, frozen in liquid nitrogen, and freeze-dried for 48 h. To prepare SA-hydrogel nanocomposite samples, different amounts of GO were suspended in deionized water (3.5 mL) using an ultrasonic bath, and then added into the mixture with acrylic monomers. According to the aforesaid procedure, different SA-g-poly(AAm-co-GMA) hydrogel samples (with or without GO) were synthesized by changing the precursor’s weight ratios (Table 1).

**Table 1:** Different SA-based hydrogel samples prepared via changing feeding ratios of GMA monomer, MBA cross-linker, and GO nano-filler and their water absorbencies.

Entry	GMA/alginate (v/w%)	GO/alginate (w/w%)	MBA/alginate (w/w%)	WA (g/g)
A1	0.1	0	6	1.3
A2	0.02	0	6	3.2
A3	0.01	0	6	3.3
A4	0.01	4	0	3.4
A5	0.01	4	1	3.7
A6	0.01	4	2	3.1
A7	0.01	4	4	2.3
A8	0.01	0	2	17.9
A9	0.01	1.2	2	7.7
A10	0.01	2.4	2	4.1

### Synthesis of CH-based hydrogels

CH-based hydrogel samples were synthesized by first dissolving chitosan (3 g), in 100 mL of acetic acid solution (2%). Next, acrylamide (75 mg), and certain amounts of GMA were dissolved in 4 mL deionized water and added to the chitosan solution (5 mL). The reaction was stirred for 5 min to prepare a homogenous mixture, and then MBA was added to the reaction flask. The next steps were similar to the procedure used for SA-based hydrogels, which after 15 min stirring, APS (5 mg) was added to the mixture and it heated up to 80 °C, for initiating the radical polymerization reaction. The as-synthesized gels were swelled in deionized water for 24 h. Then they were separated from water, frozen in liquid nitrogen, and freeze-dried. Similarly, for the preparation of nanocomposite samples, different amounts of GO were suspended in deionized water (1 mL) and added into the mixture in the step that acrylic monomers were added. Table 2 showed the different CH-g-poly(AAm-co-GMA) hydrogel samples synthesized by changing the precursor’s weight ratios.

**Table 2:** Different CH-based hydrogel samples prepared via changing feeding ratios of GMA monomer, MBA cross-linker, and GO nano-filler and their water absorbencies.

Entry	GMA/chitosan (v/w%)	GO (w/w%)	MBA/chitosan (w/w%)	WA (g/g)
C1	0.01	0	5	15.7
C2	0.01	0	10	14.2
C3	0.01	0	15	12.3
C4	0.015	0	5	11.9
C5	0.02	0	5	9.1
C6	0.02	0	10	16.3
C7	0.025	0	10	8.7
C8	0.02	2	10	9.5
C9	0.02	4	10	6.8
C10	0.02	8	10	5.4

### Characterizations

Fourier-transform infrared spectroscopy (FTIR, Thermo spectrometer), thermogravimetric analysis (TGA, TA Instrument; model SDT Q600), field emission scanning electron microscopy (FESEM, TESCAN MIRA II microscope), X-ray diffraction (XRD, Philips PW1730), Zeta potential measurements (NanoBrook Pals Zeta Potential Analyzer), and nuclear magnetic resonance (NMR, 300 MHz Bruker Avance-III 300) have been utilized to characterize the hydrogel samples.

### Water absorbency of hydrogel samples

The dried hydrogels were weighed and swelled in deionized water at room temperature for 1 h. Then, the swollen gels were filtered and weighed again. The water absorbencies [WA (g/g)] were calculated by Eq. (1).

1 $$WA \left(\frac{g}{g}\right)=\frac{{W}_{2}-{W}_{1}}{{W}_{1}},$$

where W_1_ was the sample weight before, and W_2_ was the sample weight after swelling in water. Furthermore, the WA values of samples were measured in different saline solutions (0.001 M of NaCl, CaCl_2_, and FeCl_3_), and different pHs (i.e. 3.0 to 9.0) using Eq. (1), at room temperature.

### Enzyme immobilization

PersiXyn9 was in-silico screened from the cow rumen metagenomic data and cloned, expressed, and purified in Agricultural Biotechnology Research Institute of Iran (ABRII). Sequence data of PersiXyn9 have been deposited in the GenBank (OK235706). The theoretical isoelectric point (PI) of the enzyme was computationally determined (6.5), using the Expasy Compute pI/Mw^30^.

The enzyme was immobilized on the hydrogel samples via swelling the dried hydrogel in the enzyme solution medium. Finding the optimum conditions of immobilization for each sample, different enzyme solutions (300 µL, in phosphate buffer 50 mM, pH 3.0 to 8.0) were prepared and then added separately to different hydrogel samples, in a microfuge tube and let to fully swelled, at room temperature.

### Effect of hydrogel carriers on the activity and stability of the immobilized enzyme

To estimate the activities of the free and immobilized PersiXyn9 on different hydrogel supports, 60 μL of 1% solubilized birchwood xylan solution was firstly added to 20 μL enzyme solution^31^. 3,5-dinitrosalicylic acid (DNS, 120 μL) reagent was added to the mixture and then incubated in a boiling water bath for 5 min. Next, the absorbance of each sample was measured at 540 nm, using a UV–Vis spectrophotometer. One unit of xylanase activity was defined as the amount of enzyme that released 1 micromole of corresponding reducing sugars per minute per milliliter under the assay condition^32^.

Finding the effect of temperature on the activity of the free and immobilized enzyme on each hydrogel sample, the enzyme activities were measured after incubating for 60 min, at different temperatures (40 to 80 °C). To evaluate the catalytic performance and storage stability of the immobilized enzyme on each hydrogel sample, the free enzyme and four immobilized samples were incubated at their optimum pH and temperature conditions for 0.5, 1, 2, 3, and 4 h. Next, the birchwood xylan (1%w/v) was added to the reaction mixtures, followed by the activity measurement of the enzyme at the optimum condition^25^. For enzyme leaching studies, the enzyme was separately immobilized on each hydrogel carrier at optimal conditions, and then the immobilized samples were incubated at various temperatures (40 to 70 °C, for 1 h) and different times (30 to 180 min, at 50 °C). Next, samples were washed with phosphate buffer (50 mM, pH 6), and the leached enzyme concentration in the buffer solution were analyzed^25^. In the reusability study, the residual activity of the immobilized PersiXyn9 on each hydrogel sample was measured by reusing it for 12 consecutive reaction cycles, at pH 6 and 50 °C. In each recovery run, the immobilized enzyme was incubated with the substrate, and then it was separated from the reaction mixture using centrifugation. Next, the precipitates were washed with phosphate buffer (50 mM, pH 6), and then added to fresh birchwood xylan (1%w/v) solution. The specific activity calculated in the first cycle was considered 100%.

## Results and discussion

### Synthesis of two different hydrogel support samples

#### SA-based hydrogel support samples

**Figure 1:**
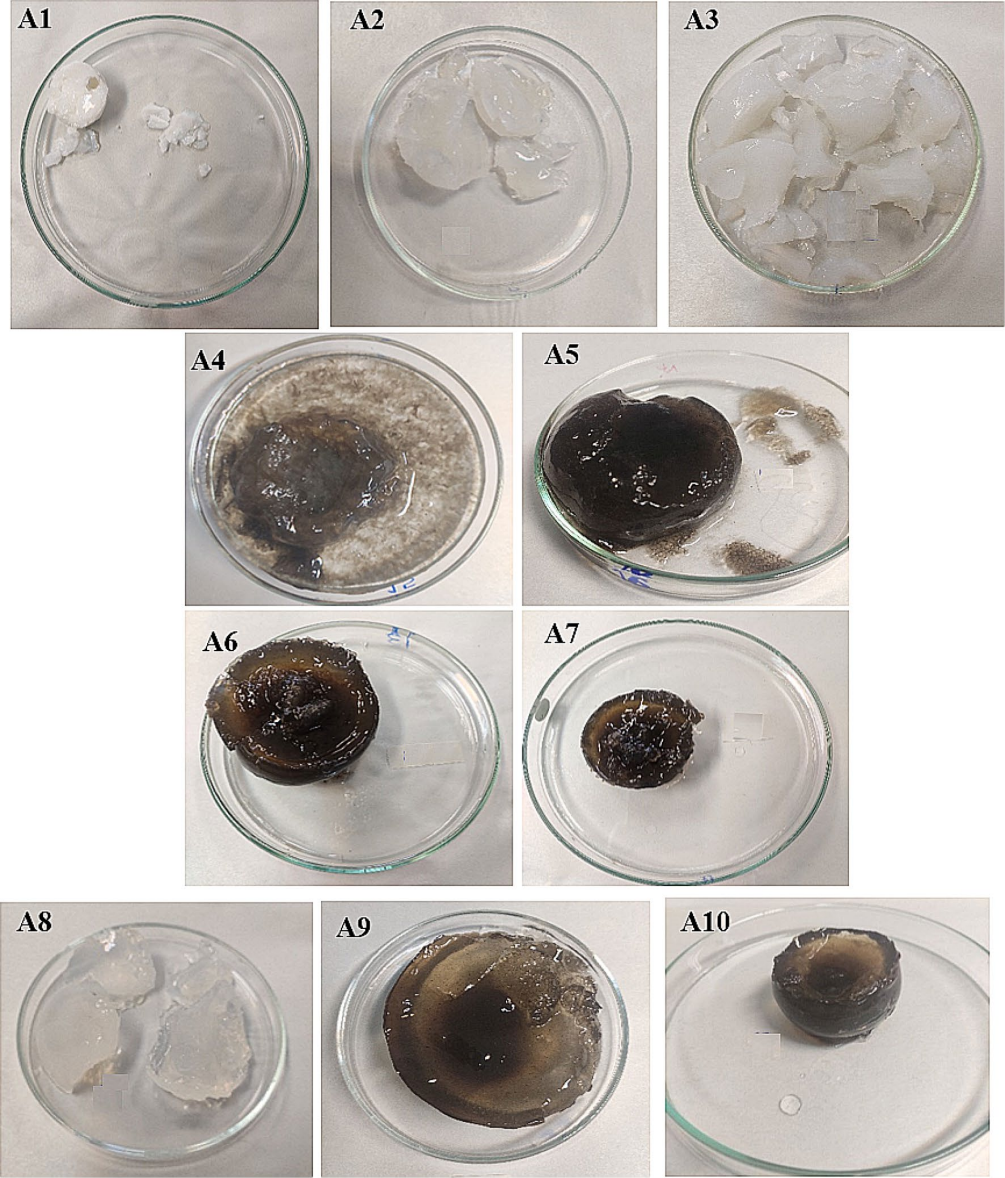
Photos of different SA-based hydrogel samples swelled in deionized water before drying process.

In the first step, to explore the effect of precursor amounts and nano-filler dosages on the properties of the synthesized hydrogels, the content of biopolymer and acrylamide remained constant. Then, different samples were prepared with changing the contents of MBA, GMA, and GO in the polymerization reaction, for both SA- and CH-based hydrogels (Tables 1, 2). Subsequently, water absorbencies of the as-synthesized samples were evaluated at different conditions. In the case of SA-based hydrogels, it was found that the amount of GMA could play a crucial role in the water absorbency and the network-gel strength of the samples (Table 1, and Fig. 1, entry A1–A3). The water absorbency was increased by decreasing the GMA content (i.e. 0.1 to 0.01 v/w%), from 1.3 to 3.3 g/g. This might be hypothesized that highly cross-linked rigid hydrogel networks were synthesized at higher dosages of GMA, which caused limited expansion of the gel for storing a large amount of water^33^. Then, the amount of GO nano-filler and GMA were fixed and the water absorbencies of the hydrogels prepared with different amounts of MBA were evaluated (Table 1, and Fig. 1, entry A4–A7). The results revealed that to prepare the stable and well-structured SA-based hydrogel nanocomposite with appropriate swelling behavior, using the chemical cross-linker was obliged and without MBA the 3D network of gel could not be formed (Fig. 1, A4). Furthermore, the water absorbencies were found to be 3.4, 3.7, 3.1, and 2.3 g/g, for A4, A5, A6, and A7 samples, respectively. Similar to GMA content, in higher amounts of chemical cross-linker (> 1 w/w %), the formation of hydrogel with a high cross-linked and rigid network might result in a decrease in WA values. Finally, the effect of the GO amounts on the SA-based hydrogel properties was investigated. For this reason, different hydrogel samples were synthesized at optimum dosages of MBA (2 w/w %), and GMA (0.01 w/w %), with various GO contents (Table 1, and Fig. 1, entry A6, A8–A10). The measured WA values for these samples displayed that the swelling capacity of hydrogels was continuously decreased with increasing the content of the GO (Table 1, entry A6, A8–A10). For instance, sample A6, with the highest amount of GO, showed the lowest WA value (3.1 g/g), while sample A8, which was prepared without GO, had the greatest WA value (17.9 g/g). The significant effect of GO dosages on the gel strength and structure could be also observed in the photos of these samples before the freeze-drying process. Such inverse relation between GO content and swelling capacities of the bio-based hydrogels has been reported in the previous studies^27^. This suggested the role of GO nano-sheets as physical cross-linker in the hydrogel matrixes which resulted in the formation of more rigid gels with lower WA values. For more explanation, the reinforcement mechanism of polymer nanocomposites could be justified through the favored interfacial interactions, such as hydrogen bonds, electrostatic attractions, and Van der Waals forces, between polymer and nano-filler^34^. In other words, the homogenous distribution of nanomaterials in the hydrogel matrixes without the addition of any surfactants/stabilizers portrayed the importance of functional groups in both host/guest components in the formation of such favored interactions^35^. For both SA- and CH-based hydrogel nanocomposites, the as-mentioned physical interactions between the ionic moieties on the side chains of the polymers and oxygenated functional groups on the surface of GO nano-sheets could govern the physical linking of the flexible polymeric chains by GO, and consequently led to the reinforced hybrid polymer. This effect has been reported previously for hydrogel nanocomposites synthesized using GO, metallic nanoparticles, or cellulose nanocrystals as nano-filler^36–38^. The decreased swelling capacities of nanocomposite samples compared to the neat hydrogels could be attributed to the fact that the mobility of the polymeric chains was restricted in the reinforced nanocomposite. In the presence of GO, more potential junctions were formed between polymeric chains, which adversely affected the water absorbency of nanocomposite samples.

**Figure 2:**
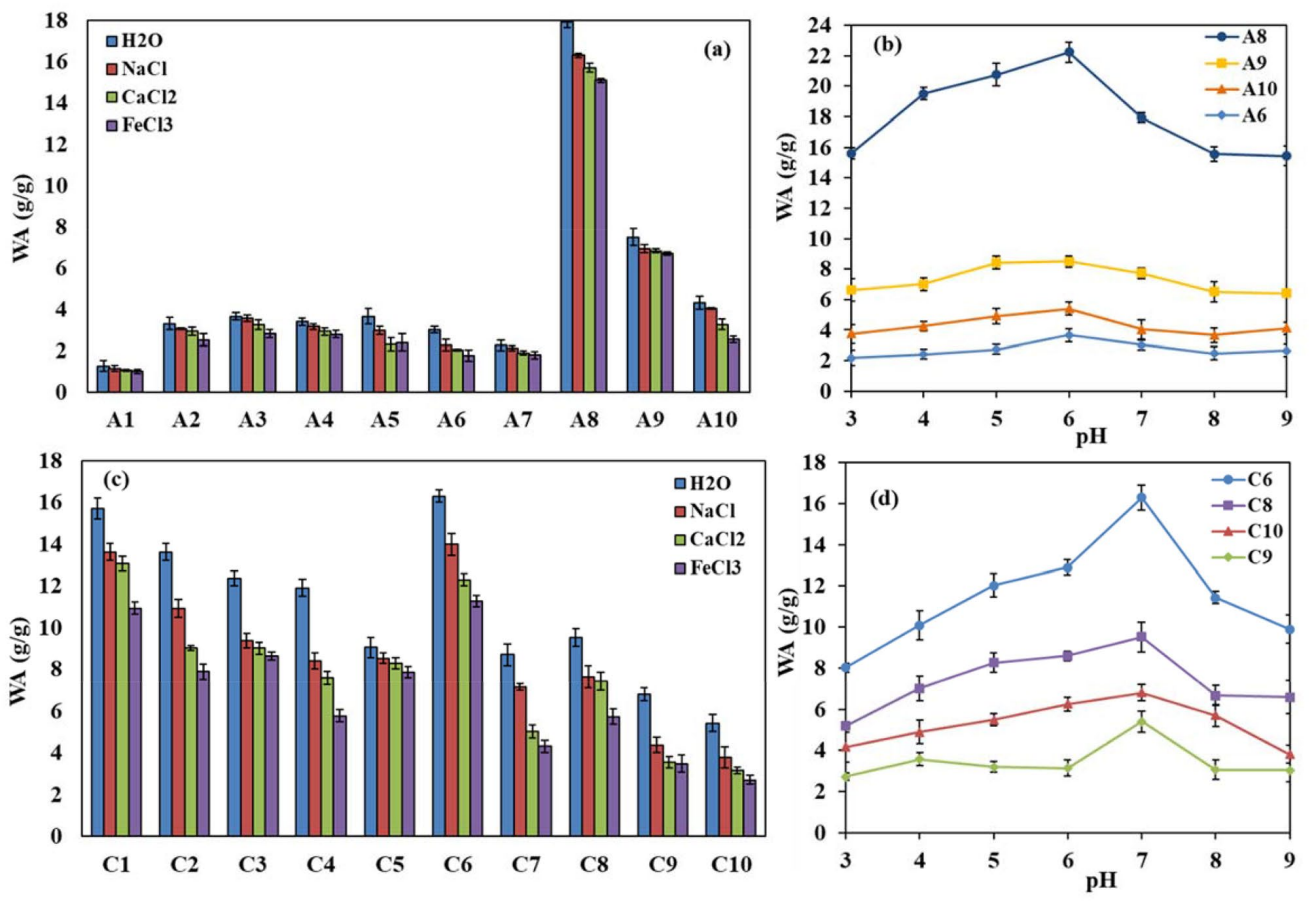
Effects of different (**a**) salt solutions, and (**b**) pHs on the water absorbency of the as-synthesized SA-based hydrogel samples; effects of different (**c**) salt solutions, and (**d**) pHs on the water absorbency of the as-synthesized CH-based hydrogel samples.

Moreover, the water absorbencies of the SA-based hydrogel samples were estimated at different pHs and salt solutions (Fig. 2). As expected, all the samples showed higher water absorbencies in deionized water than saline solutions. The decrease in differences between osmotic pressure in the salt solutions and the swelled hydrogel was declared as a reason for the decrease in WA values in the presence of salts^39^. Besides, the swelling capacities of sample A8 (pristine SA-based hydrogel without GO), in all saline media, were much higher than other samples (Fig. 2a). As mentioned before, the incorporation of GO in the matrix of the hydrogels had a negative effect on their water absorbencies in deionized water and also in saline solutions. Furthermore, a comparison of the WA values of the hydrogel nanocomposite samples (A6, A9, A10) with pristine hydrogel (A8), at different pHs revealed their almost similar trend (Fig. 2b). All the samples were swelled little at acidic pH of 3, but WA values were enhanced by increasing the pH up to the pH of 6, and then they were decreased in the higher pHs (Fig. 2b). All hydrogel samples containing GO (A6, A9, and A10) showed a slighter dependency on pH than pristine hydrogel (A8). It was approved that at acidic pHs, the protonation of functional groups on the polymeric chains caused decreasing the swelling capacities of the hydrogels. Conversely, at neutral pHs, deprotonation of the functionalities in the hydrogel resulted in the anion–anion repulsions and higher WA values. In the alkaline media, the presence of Na ions (from NaOH) led to the shielding of the functional groups of the polymeric chains, and restriction of anion–anion repulsions, which consequently decreased the swelling capacities of samples ^27,40^.

#### CH-based hydrogel support samples

The swelling capacities of CH-base hydrogel samples were generally higher than SA-based hydrogels, and again, in this case, the water absorbencies and the network-gel strength of the samples were notably dependent on GMA, MBA, and GO amounts (Table 2). The water absorbencies of these samples were increased by decreasing the GMA content. For instance, changing the GMA content from 0.02 to 0.015, and 0.01 v/w%, at constant MBA and GO amounts, resulted in the samples with WA values of 9.1, 11.9, and 15.7 (g/g), respectively (Table 2, entry C1, C4, C5). Again, the increase in the dosage of MBA could not positively change the WA properties of the CH-base hydrogels (Table 2, entry C1–C3). Interestingly, it was found that at GMA content of 0.02 v/w%, and MBA dosage of 10 w/w%, a hydrogel sample with the highest WA value (16.3 g/g) was synthesized (Table 2, entry C6). It could be hypothesized that there was an optimal ratio between the dosage of MBA and GMA in the hydrogel formulation, and when this ratio was kept, the amount of water absorption reached its maximum. Next, the best precursor amounts to synthesis pristine CH-based hydrogel with the optimum water absorbency were selected from entry C6, and then at this condition, the content of GO nano-filler was changed to prepare hydrogel nanocomposite samples (Table 2, entry C6, C8–C10). Similar to SA-based hydrogels, the WA values of CH-based samples displayed that their swelling capacities were decreased with enhancing the content of GO. For instance, with increasing the GO dosage from 2 to 4 and 8 (w/w%), the WA values were measured to be 9.5, 6.8, and 5.4 g/g, respectively. These results again confirmed the role of GO nano-filler as a physical cross-linker in the network of the bio-based hydrogels.

In the following, the water absorbencies of the CH-based hydrogels were estimated at different pHs and salt solutions. In contrast to A-family samples, the WA values of the different CH-based hydrogels were not extremely different from each other in saline solutions. Again, water absorbencies of all the samples were higher in deionized water than in saline solutions (Fig. 2c). It could be seen clearly that the pristine CH-based hydrogel (C6) had a much higher WA value than the hydrogels containing GO (C8–C10), in all saline solutions. Then, the swelling capacity of C6 was compared with those of the C8–C10 samples, at different pHs (Fig. 2d). The results showed that sample C6 had the highest WA values in all pH media, but for all the samples, the optimum pH for maximum swelling capacity was 7.

According to the WA results, two candidates from each family were chosen for the rest of the experiments during this study which were A8, and A9 from SA-based hydrogels, and C6, and C8 from CH-based hydrogels. Utilizing these samples, it would be possible to examine the effects of biopolymer and GO nano-filler on the characteristics of the hydrogel samples and their enzyme immobilization performance. To explain the importance of water absorbency as the index of selecting synthesized hydrogels for the following experiments, the relationship between water absorbency and enzyme immobilization efficiency should be considered. One of the imperative factors in controlling the interactions between enzymes and hydrogels is the water absorbency of the hydrogel supports. Higher swelling capacities result in free movements of the polymeric chains in the enzyme solution medium and make the soft gels. According to our previous studies, hydrogel samples with the higher WA values could entirely swell and reach their maximum absorbance capacity even at the lowest amounts to show maximum enzyme immobilization efficiencies (100%)^27^. Conversely, in the case of the hydrogel samples with lower WA values, higher amounts of the sample should be used to form a soft gel in the enzyme solution and achieve 100% immobilization efficiency^27^. So the enzyme-hydrogel interactions are increased, and higher enzyme entrapment is possible in the flexible water-saturated gels due to the unrestricted mobility of their polymeric chains. These finally led to higher immobilization efficiencies, and consequently, higher amounts of the enzymes were entrapped in the support network. Because the enzyme activity is dependent on the enzyme amount, it may cause enhanced relative activities of the enzyme. Besides water absorbency, it should be noticed that microstructure and network charges of the hydrogels have played key roles in their enzyme immobilization performance which will discuss in the following sections.

### Characterization of as-synthesized hydrogel supports

^1^H-NMR spectroscopy in D_2_O and FTIR analyses were utilized to confirm successful grafting of the biopolymer backbone to AAm and GMA monomers. The ^1^H-NMR spectrum of SA-g-poly(AAm-co-GMA) hydrogel showed several new resonance peaks compared to the spectrum of neat sodium alginate indicating different species of H appeared after the polymerization reaction (Supplementary Fig. S1). Particularly, the peaks at chemical shifts of δ = 1.3 to 2.4 ppm approved the methylene and methane protons of grafted monomers which was effectively attached to alginate polymer.

FTIR spectrum of A8 sample showed the strong and broadband at 3421 cm^−1^ corresponding to the stretching vibrations of –OH groups of SA polymer (Supplementary Fig. S2). The peak at 2932 cm^−1^ was originated from stretching vibrations of –CH_2_ groups on the SA backbone and the grafted AAm and GMA polymeric chains. The characteristic bands at 1667 and 1415 cm^−1^ were assigned to the stretching vibrations of –C=O and C–N bonds, respectively, on the CONH_2_ functionalities, confirming the grafting of AAm on the SA polymer. The peak at 1614 cm^−1^ came from the asymmetric stretching vibrations of –COO^¯^ groups on the SA polymer. In addition, the characteristic band at 1035 cm^−1^ corresponded to the C–O stretching in the C–O–C groups of SA, and the band at 1093 was assigned to the C–O bond of aliphatic C–OH vibrations^41,42^. In comparison with neat SA-based hydrogel, the reinforced sample with GO (A9) displayed characteristic peaks at lower wavenumbers which could be a result of possible hydrogen bonds formed among the hydroxyl groups of SA and GO nano-sheets^43^ (Supplementary Fig. S2). However, the low content of GO in the polymer matrix caused its weak characteristic peaks could not be observed in the FTIR spectrum of the A9 sample. The spectrum of the C6 sample showed similarly a broad absorption peak around 3430 cm^−1^ assigned to O–H and N–H groups. The peaks at 2930 and 1669 cm^−1^ have corresponded to the stretching vibrations of –CH_2_ and –C=O groups of the grafted AAm chains, respectively. The characteristic bands at 1557, 1409, and 1072 cm^−1^ were originated from bending vibrations of N–H (in the primary amine groups) and –CH_2_ groups, and asymmetric stretching vibrations of the C–O–C bridges, respectively (Supplementary Fig. S2)^43^. Again, the FTIR spectrum of the neat CH-based hydrogel differed from the reinforced sample with GO (C8) just by little shifts of the characteristic peaks toward lower wavenumbers (Supplementary Fig. S2).

XRD analyses have been utilized as a supplementary technique for the determination of the GO in the matrix of the hydrogels. The XRD patterns of all hydrogel samples indicated their amorphous morphology with a broad diffraction peak at 2Ө around 20°–22° (Supplementary Fig. S3). Interestingly, compared to the neat hydrogel samples (A8, C6), the samples containing GO (A9, C8) showed a weak signal appeared at 2Ө = 10°. This diffraction peak could be originated from the diffraction of (0 0 2) layer planes of GO and confirmed the formation of hydrogel nanocomposites.

**Figure 3:**
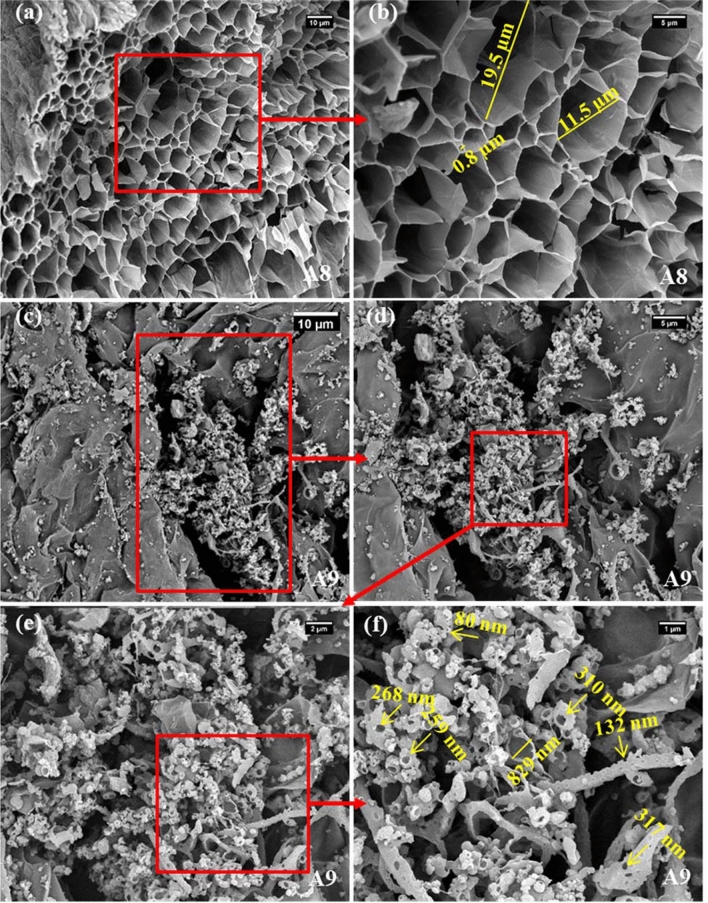
SEM images of selected SA-based hydrogels (**a**,**b**) A8, and (**c**–**f**) A9 samples.

SEM analyses were utilized to observe the interior microstructure and morphology of the hydrogel samples. SEM images of A8, clearly showed the polyporous structure of this sample, with an interconnected macropores framework. The pore diameters varied between 0.8 and 20 µm, and such a hollow and integrated porous network provided a great surface area and possibly facilitated easy access for enzyme immobilization (Fig. 3a,b). Interestingly, the morphologies of the samples were intensely affected by the existence of the GO nano-sheets in the matrix of the hydrogel. SEM images of A9 displayed a quite different microstructure from the A8 sample. At low magnification, the structure of the A9 hydrogel sample was piled up with non-uniform stacked globes and rod shape particles (Fig. 3c,d). An enlarged view of this sample structure exhibited opened cocoon-like particles combined with the distorted capillaries containing abundant holes in which pore diameter differed from 80 to 800 nm (Fig. 3e,f). It could be hypothesized that the addition of GO in the gel formulation has resulted in the formation of hydrogen bonds and electrostatic attraction between SA backbone, PMA, and GMA polymeric chains, which made entangled and non-uniform structures without distinct channels like the A8 sample.

**Figure 4:**
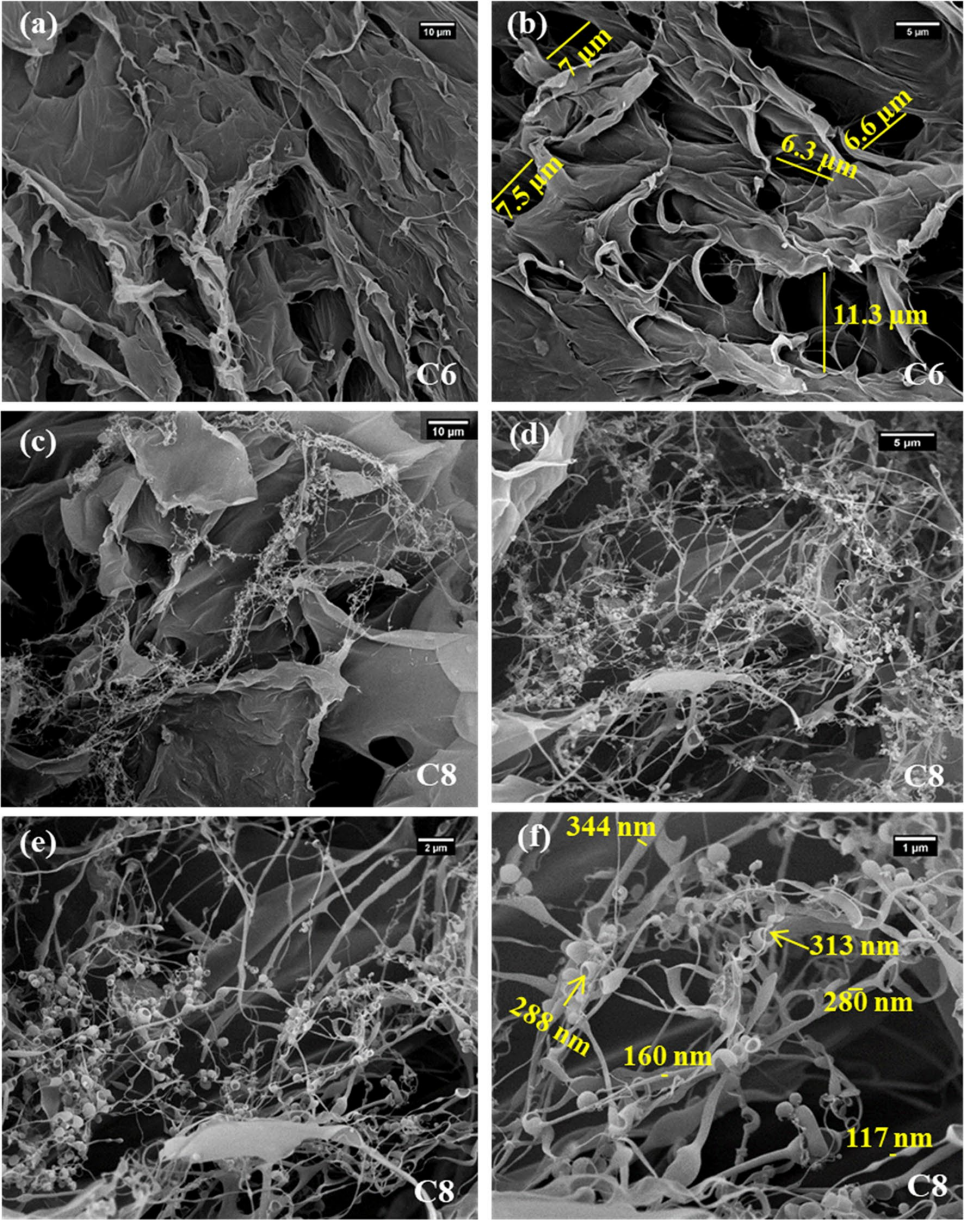
SEM images of selected CH-based hydrogels (**a**,**b**) C6, and (**c**–**f**) C8 samples.

Comparison of the SEM images of A8 and C6 samples showed that the microstructure and morphology of the hydrogels could be entirely controlled by its biopolymer backbone. As one can see clearly, in contrast to SA-based hydrogel, the sample synthesized using chitosan biopolymer did not show ordered interconnected cell architecture (Fig. 4a,b).

**Figure 5:**
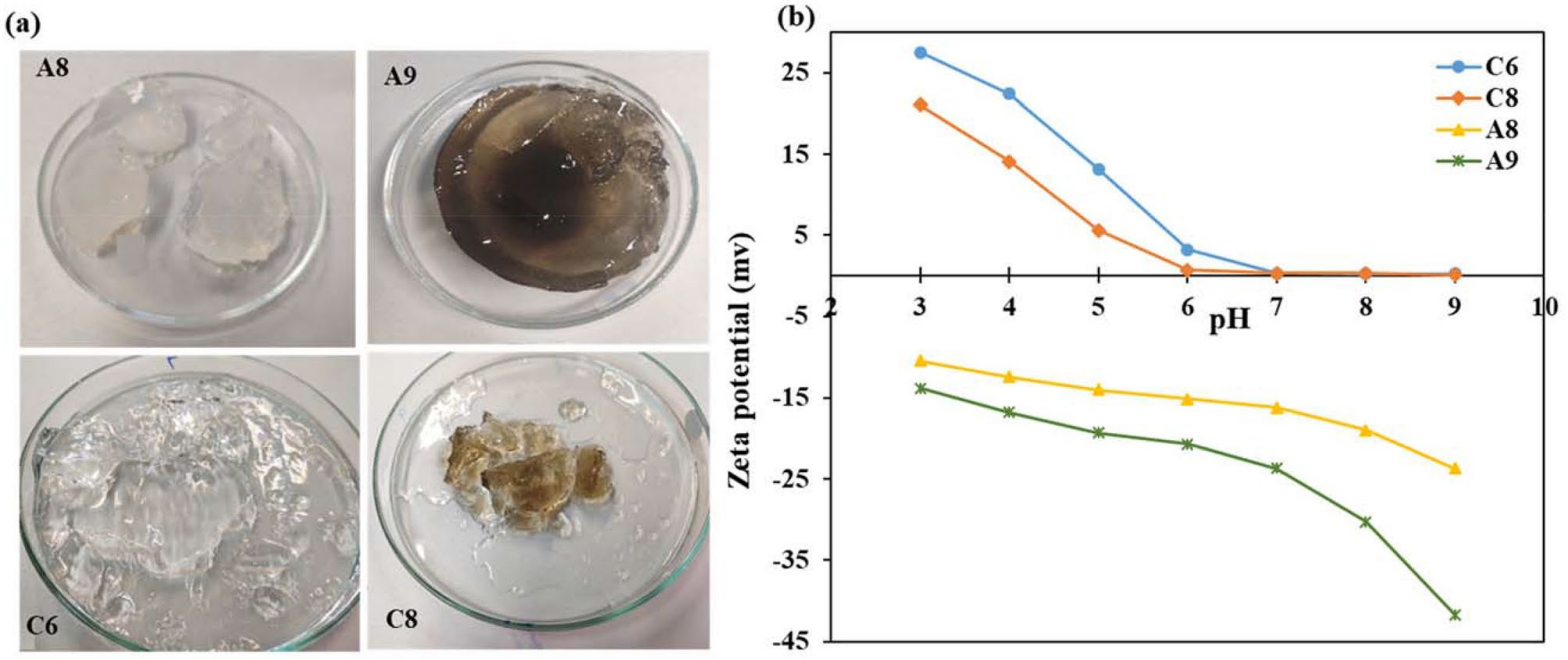
(**a**) Photos of the selected SA-based (A8, A9), and CH-based (C6, C8) hydrogel samples swelled in deionized water before drying process. (**b**) Zeta potential analysis of selected SA-based (A8, A9), and CH-based (C6, C8) hydrogels.

In the SEM images of the C6 sample, there were macro-pores with an average diameter ranging from 6 to 11 µm (Fig. 4b). It seemed that the gel structure was collapsed during the freeze-drying process and distinct cells with exact wall boundaries could not be formed in this sample. It could be attributed to the fact that CH-based hydrogel samples formed very soft and malleable gels during water absorption possibly due to their lower cross-linking degrees. Similar to SA-based samples, the addition of the GO in the matrix of the CH-based hydrogels led to significant changes in their morphology and architecture. SEM images of the C8 sample revealed many filaments and fibers which were connected to clew-like globes (Fig. 4c–f). The thickness of the filaments varied from 100 to 350 nm, and the pore diameter of cocoons was around 300 nm. Converse to the A9 sample, in SEM images of the C8 hydrogel the population of fibers is more abundant than open cocoons. Moreover, the macrostructure and gel softness of the selected hydrogel samples were compared and contrasted using their photos (Fig. 5a).

Finally, the effects of pH (3.0–9.0) on the zeta potential (mV) of the hydrogels were evaluated for A8, A9, C6, and C8 samples. As expected, the pH of the medium controlled the surface charge of the samples, and besides the ζ-potential values were governed by the type and charge of the biopolymer which was utilized for the synthesis of the samples as well as the content of the GO nano-sheets. While SA-based hydrogels showed negative charge within all the examined pH ranges, but CH-based samples displayed positive or neutral charges at different pH solutions (Fig. 5b). In a range of pH 3.0–9.0, the zeta potential values changed between + 27.5 and + 0.1 mV, for C6 sample, and between + 21.5 and + 0.1 mV, for C8 sample. In all the pH solutions the C6 sample showed more positive charges which confirmed that the GO addition in the formulation of the hydrogel may induce a negative charge to the polymer matrix due to its negative oxygen functional groups. When pH of the solution increased up to 6.0, the ζ-potential results for both samples showed ⁓ 0.1 values which are considered neutral ranges. This indicated the dominant effect of the positive charge of the chitosan biopolymer in the hydrogel matrix which could cover the ionization of carboxyl groups in these samples even at basic pHs. In fact, chitosan as polycation with a reported pKa of 6.50 contains amino groups that could easily be positively charged, so at acidic pHs, the protonation of amino groups caused a highly positively charged hydrogel network, which turned neutral at the pHs above 6.0^44^. Conversely, both SA-based samples displayed negative charges at different pH ranges which were from − 10.4 to − 23.7 mV, for the A8 sample, and from − 13.8 to − 41.7 mV, for the A9 sample. Again, the existence of GO nano-sheets in the hydrogel network resulted in the more negatively charged polymer. Therefore, it would be expected that using sodium alginate as anionic polysaccharide with pKa around 3.5 in the hydrogel formulation resulted in the negatively charged gel network in all examined pH ranges. For more explanation about the importance of measuring zeta potential, it should be stated that ζ-potential values of carriers are particularly worthy in the physical immobilization enzymes on the hydrogels. In such cases, robust immobilization was provided through effective electrostatic attractions between the enzyme and its carrier. The PI of PersiXyn9 was calculated to be 6.5. Hence, when SA-based hydrogels were swelled in the buffer solution containing enzyme at pH 6, the charge of the protein was positive, and the net charge of the hydrogel support was negative (Fig. 5b). Considering this point, it could be expected that efficient electrostatic attractions were involved in the immobilization process, which guaranteed the preparation of stable PersiXyn9/SA-based hydrogel conjugates. Conversely, the zeta-potential of CH-based hydrogels showed an almost neutral charge at this pH condition, and consequently superb electrostatic interactions could not be expected between enzyme and these chitosan supports. The effects of the hydrogel support’s charges and structures on the enzyme immobilization are discussed in detail in the next section.

### Enzyme immobilization

**Figure 6:**
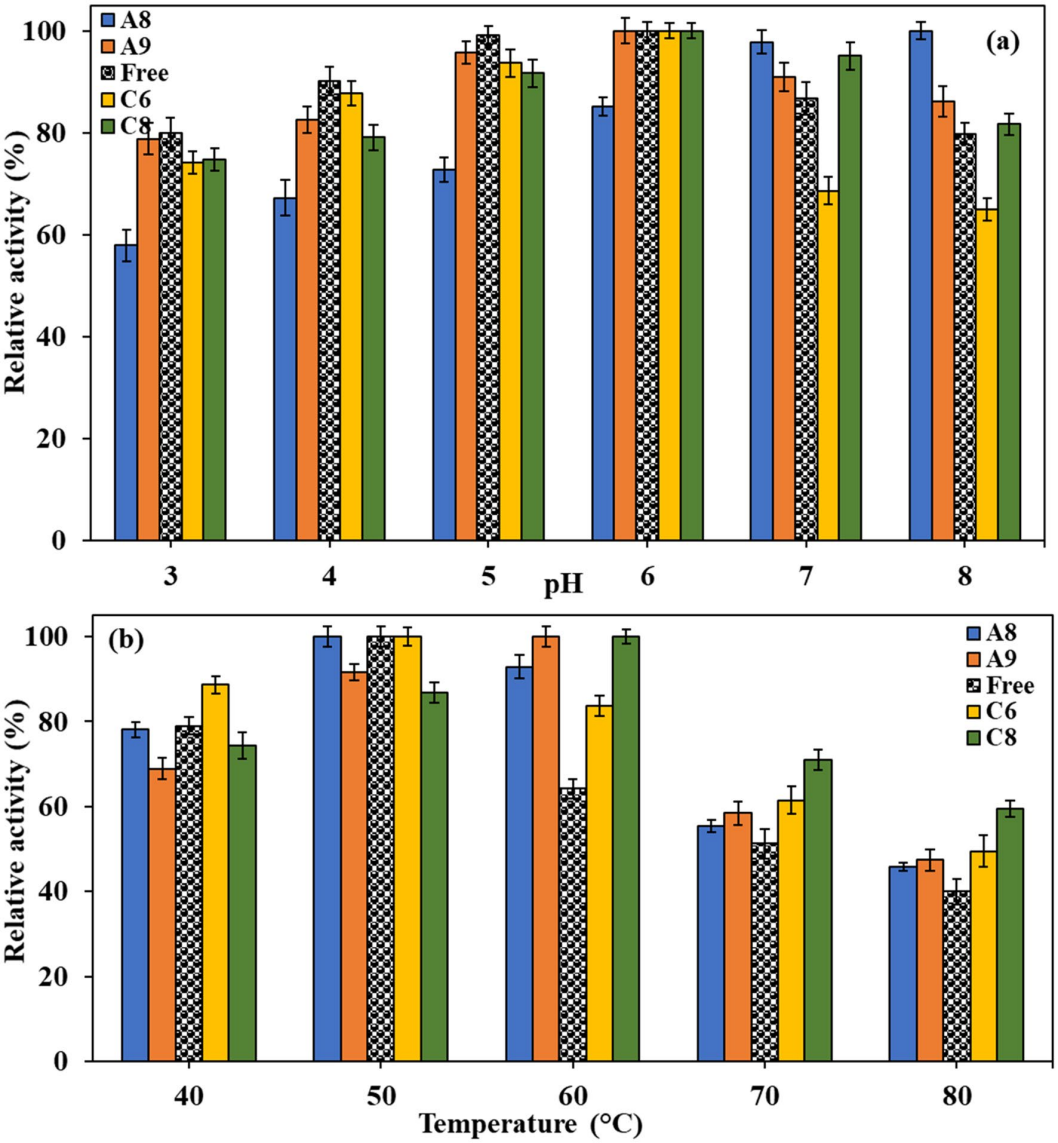
Effect of (**a**) pH and (**b**) temperature on the activity of the free and immobilized PersiXyn9 on the selected hydrogel supports.

In the first step, the optimum pH of the immobilized and free enzymes was investigated (Fig. 6a). The free PersiXyn9 showed more than 80% activity at all of the examined pHs (3.0 to 8.0), which confirmed a wide range of applications for industrial utilization of this enzyme. While the maximum relative activity (100%) for the free enzyme was estimated to be at a pH range of 5–6, the optimum pH value for the immobilized PersiXyn9 on the A9, C6, and C8 were found to be at a pH of 6.0. The immobilized enzyme on the A8 sample showed the optimum pH (100% activity) of 8.0. Interestingly, free PersiXyn9 displayed a decrease in its activity at pH values higher than 6.0, but upon immobilization on C8 and A9 support samples, such negative effects of alkaline solutions on the enzyme activity were somewhat retarded. For instance, free enzyme retained 79.8% of its maximum activity at a pH of 8, which this value reached by the enzyme immobilization on C8 and A9 hydrogels to 81.7%, and 86.1%, respectively. Therefore, it would be suggested that immobilization, particularly on the A8 sample, could provide a new practical application of PersiXyn9 which required working at alkaline conditions.

The temperature profiles of the free and immobilized PersiXyn9 were evaluated by fixing the pH of each sample at its optimum pH, and measuring the enzymatic activities at a temperature range between 40 to 80 °C. While the optimum temperature for the free enzyme and its immobilized forms on the A8 and C6 supports was 50 °C, it was shifted to a higher temperature (60 °C) in the case of the immobilized enzyme on A9 and C8 supports (Fig. 6b). At high-temperature ranges between 60–80 °C, all the immobilized forms of the PersiXyn9 demonstrated higher enzyme activities. For instance, the relative activities were estimated to be 92,9%, 100%, 83.6%, 100%, for the immobilized enzyme on the A8, A9, C6 samples, respectively, which were higher than the free enzyme activity (64.2%), at 60 °C.

There is a reasonable concern about enzyme immobilization on polymeric supports which pointed to loss in the catalytic activity of the enzymes due to the mass transfer restrictions for substrate “in” and the enzyme “out” from the carrier. Such concerns could be addressed by using cross-linked hydrogel carriers because their flexible character leads to swelling of the hydrogels in the buffer solutions. The immobilized enzymes on the hydrogels make soft and flexible gels in aqueous media enhancing mass transfer possibility and resulting in minimum loss of the catalytic activity. There are several examples in the literature where the catalytic activity of enzymes increased upon conjugation onto the hydrogels^45–48^. For example, in our previous reports on the immobilization of the recombinant xylanases on the CMC-based hydrogel, the ‘Km’ values were increased slightly upon immobilization on the carrier^25,28^. The difference in ‘Km’ values between the free and immobilized forms of the enzymes could be related to a limitation in the accessibility of substrate to the active site of the immobilized enzyme. So, these our previous data approved that a flexible hydrogel structure could provide a suitable platform for the enzyme’s active sites to have effective access to the substrate with higher affinity. From another viewpoint, there would be another concern about enzyme leaching from the gels that will be discussed in the next sections.

**Figure 7:**
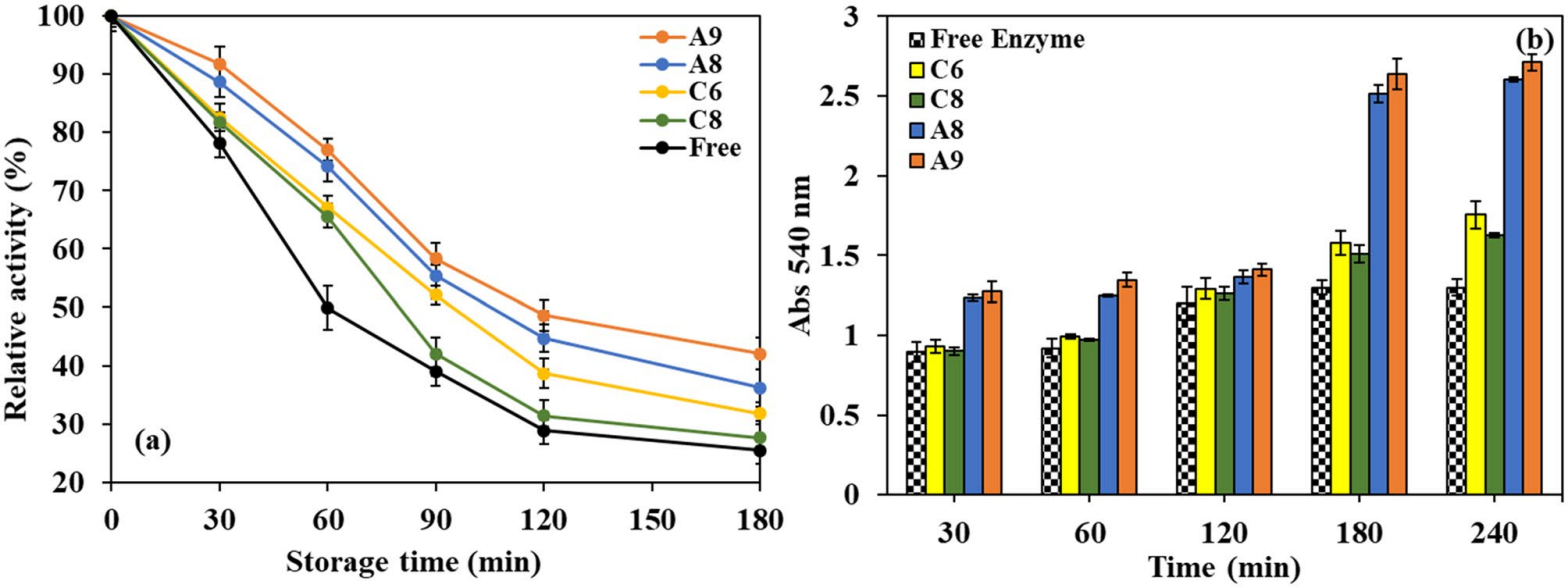
(**a**) Storage stabilities, and (**b**) comparative analysis of the reducing sugar release after treatment with the free PersiXyn9 and its immobilized forms on the selected hydrogel supports.

The storage stabilities of the free and all the immobilized PersiXyn9 were evaluated via investigation of the enzyme activities within 180 min, in different time intervals. Each enzyme sample was incubated at its optimum pH and temperature conditions. The immobilized enzyme on all the hydrogel supports showed higher relative activities than its free form (Fig. 7a). After incubation for 60 min, the activity of the free enzyme was dropped to 49.8%; however, the activities of immobilized enzymes on A8, A9, C6, and C8 samples were 74.2, 77.1 67.1, and 65.5%, respectively. Moreover, the enzyme immobilized on the SA-based hydrogels revealed higher storage stability rather than CH-based hydrogels. For instance, after 180 min, the enzyme relative activities on A9 and A8 hydrogels were 42.1 and 36.2%, respectively, but at the same condition, the enzyme activities on C8 and C6, were measured to be 31.7, and 27.6%, respectively. In both hydrogel families, the samples which contained GO showed higher storage stabilities than the neat hydrogel samples.

In the next step, the catalytic performances of the free and all the immobilized enzyme samples were analyzed via monitoring the concentration of the reducing sugars released from incubation of the birchwood xylan and enzyme samples, at different times. The results displayed that, similar to storage stabilities; all the immobilized enzymes had a higher yield of the reducing sugar than the free enzyme (Fig. 7b). Again, the immobilized enzymes on the SA-based hydrogels (PersiXyn9/A9 and PersiXyn9/A8) enhanced the released reducing sugar yield in a period of 240 h, much higher than enzymes on the CH-based hydrogels (PersiXyn9/C6 and PersiXyn9/C8). Compared to the free enzyme, the PersiXyn9/A9 revealed a 110.1% increase in the released reducing sugar from xylene after 240 h, while PersiXyn9/C8 showed an increment of 35.6%.

The results so far showed that SA-based hydrogel samples had higher performances in the immobilization of the PersiXyn9 enzyme. According to our previous studies on the immobilization of the different recombinant xylanases onto the hydrogel carriers, the key parameter in the stabilization of enzymes on these supports is the strength of the interfacial interactions between enzyme and its carrier^25,28^. As mentioned before, in the case of physical immobilization strategies through simple swelling of the hydrogels in the enzyme solutions, efficient electrostatic attractions played a key role in the enzyme entrapment efficiencies. The SA-based hydrogels had a negative net charge at the pH of 6 (pH of enzyme solution for the immobilization), and it could be deduced that effective favored electrostatic interactions could be involved between enzyme and SA-based supports, which resulted in the latter being more efficient enzyme entrapment compared to the CH-based hydrogels. To confirm this hypothesis, enzyme leaching studies were conducted after incubating the immobilized samples at different conditions and then washing them with phosphate buffer solution. The enzyme concentration in the washing solution could be considered the amount of the leached enzyme and showed the enzyme entrapment performance of each carrier (Table 3). In physical enzyme immobilization, higher temperatures or longer times lead to severe enzyme leaching. In fact, in these conditions, the enzyme-support interactions would be impaired, and consequently, more enzymes might be leached out from support. As expected, our results showed that with raising the temperature/increasing the time, the amount of enzyme in the leachate was increased continuously. Interestingly, again, SA-based supports displayed stronger electrostatic attractions at the protein-hydrogel interface and lower enzyme leaching (%) compared to the CH-based hydrogel samples. Both hydrogel families, which contained GO (A9 and C8), showed lower enzyme leaching amounts than their pristine counterparts, which could be due to the existence of GO in their matrixes. Oxygenated functional groups on the GO nano-sheets might provide additional H-bonds and ionic bridge stabilizing interactions with the enzyme resulting in lower amounts of the leached enzymes from the hydrogel nanocomposite samples. Furthermore, one may ask about the effects of the microstructure and pore sizes of the hydrogel samples, as one of the plausible contributors, in the efficiency of the enzyme entrapment. In this case, as SEM images of hydrogels showed, A8 sample had a well-ordered network structure with a pore size of 0.8–20 µm (Fig. 3a,b), but C6 sample did not show interconnected cell architecture. In contrast, some macro-pores (6–11 µm) were randomly observed in the SEM image of C6 sample (Fig. 4a,b). It could be assumed that the homogeneous cell structure of A8 sample, along with its cage-like structure, might offer a larger specific area for more efficient enzyme immobilization compared to C6 sample.

**Table 3:** Enzyme leached out (%) at various incubation temperatures/times from immobilized SA-based (A8, A9), and CH-based (C6, C8) hydrogel samples.

Incubation condition	Temperature (°C), 1 h					Time (min), 50 °C				
		40 °C	50 °C	60 °C	70 °C	30 min	60 min	90 min	120 min	180 min
Enzyme leached (%)	A8	7.8	8.2	9.6	12.7	7.4	8.2	9.6	11.8	16.3
	A9	5.6	6.6	8.8	11.4	4.2	6.6	8.2	9.06	13.2
	C6	13.0	17.3	22.1	26.2	15.93	17.3	17.96	28.7	36.6
	C8	11.4	15.8	17.2	20.5	13.8	15.8	20.06	23.5	29.9

**Figure 8:**
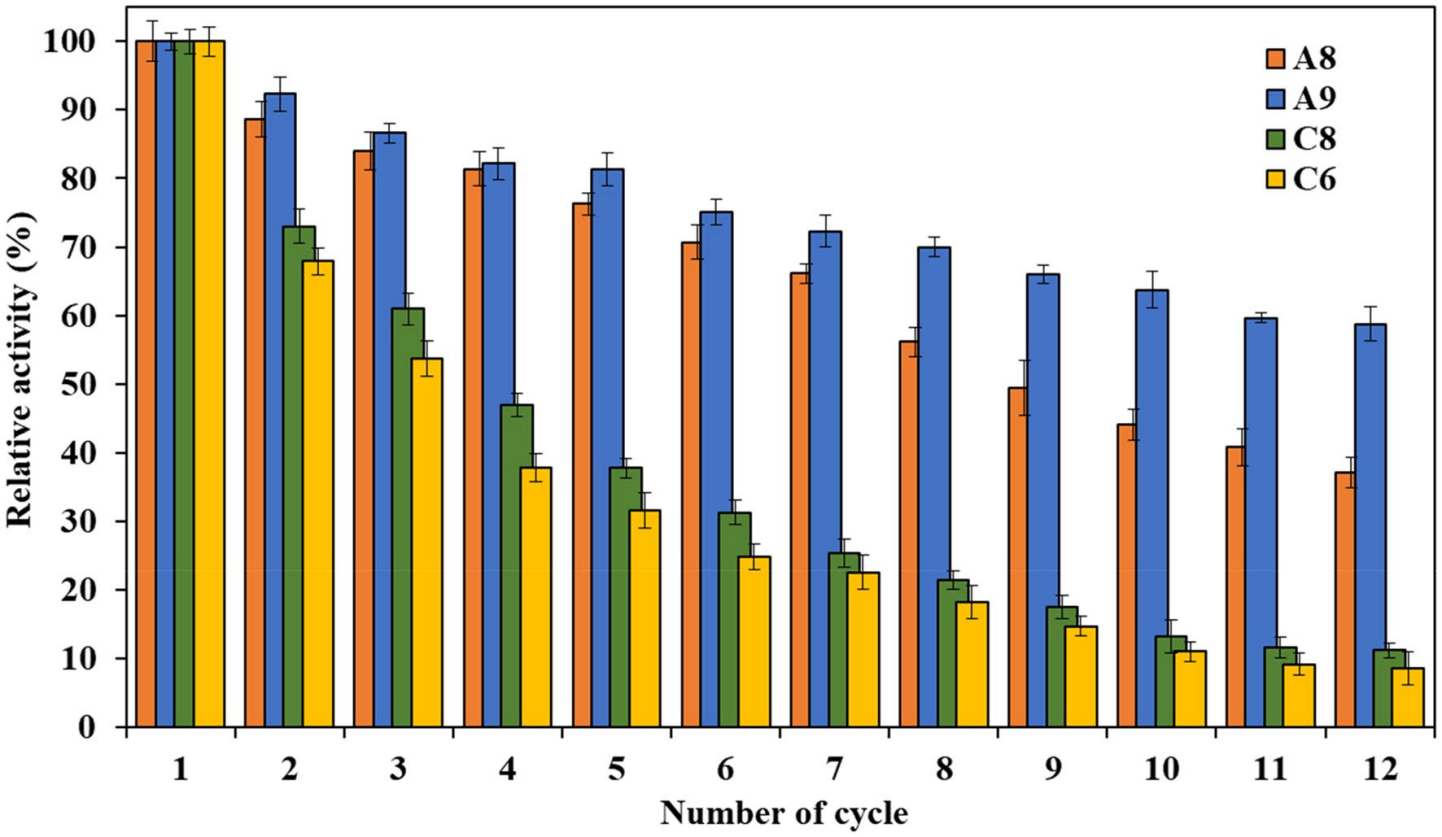
Reusability of the immobilized PersiXyn9 on the selected hydrogel supports.

The results of the reusability study demonstrated that immobilization of PersiXyn9 on the hydrogel supports could provide the reuse opportunity for the enzyme. However, a decline in enzyme activities was observed in continuous reuse cycles, which could be related to the leaching of noncovalent enzyme bindings during the incubation/washing process and distortion of protein conformation. As expected, enzyme reusability declined more in the immobilized enzyme on CH-based hydrogels than in the SA-based samples (Fig. 8). Interestingly, PersiXyn9/A9 sample could tolerate a loss of its activity during the reuse cycle better than PersiXyn9/A8. While PersiXyn9/A9 displayed 58.7% activity after twelve continuous reuse cycles, the PersiXyn9/A8, PersiXyn9/C8, and PersiXyn9/C6 retained 37.1, 11.2, and 8.5% of their initial activity at similar recovery runs, respectively.

## Conclusion

Sodium alginate- and chitosan-based hydrogels and their reinforced nanocomposites with GO have been synthesized and utilized as carriers to immobilize a model metagenomic-derived xylanase (PersiXyn9). The polysaccharide backbone of the hydrogels could control the micro-structure and net charge of the samples. Moreover, the addition of GO nano-filler not only led to significant changes in their morphology and architecture but also resulted in a decrease in their water absorption capacities. Ζ-potential values of the hydrogels were dominantly changed by the type and charge of the biopolymer. Although, the existence of GO nano-filler induced negative charges to the polymer matrixes due to its oxygenated functionalities. A comparative study of utilizing such different hydrogels as a carrier in the enzyme immobilization proved that the bio-polymer backbone had a pronounced effect on the efficiency of the samples in conjugation with enzymes. The immobilization of PersiXyn9 on all hydrogel carriers resulted in an improvement in its thermal/storage stability, along with its catalytic activity and reusability. However, the best performance has been observed for GO/SA-g-poly(AAm-co-GMA) sample. This indicated better stabilizing interactions between PersiXyn9 and SA-based hydrogel rather than CH-based samples, which confirmed the key role of electrostatic attractions between the hydrogel network and the enzyme in such physical immobilized assemblies. These findings may broaden the knowledge about relationships between the compositions of the hydrogels and their effects on the immobilized enzymes. Hence, it could be valuable in the design and development of novel and effective bio-based hydrogels for the successful engineering of optimal immobilized enzymes.

## Supplementary Information

Supplementary Figures.
