# Supplementary material for: Synthesis of two novel bio-based hydrogels using sodium alginate and chitosan and their proficiency in physical immobilization of enzymes
Source: Sci Rep. 2022 Feb 8;12:2072. doi: 10.1038/s41598-022-06013-0 (PMC8827098; doi:10.1038/s41598-022-06013-0)
Supplement: Supplementary file 1 — Supplementary Figures. [file 41598_2022_6013_MOESM1_ESM.docx]

**Synthesis of two novel and efficient bio-based hydrogels using sodium alginate and chitosan and their applications in physical immobilization of enzymes**

Fateh Shakeri^a^, Shohreh Ariaeenejad^b^, Marzieh Ghollasi ^a^*, Elaheh Motamedi ^c^*

^a^Department of Cell & Molecular Biology, Faculty of Biological Sciences, [Kharazmi University, Tehran](https://scholar.google.com/citations?view_op=view_org&hl=en&org=6837845571097102989), Iran

**^b^** Department of Systems and Synthetic Biology, Agricultural Biotechnology Research Institute of Iran (ABRII), Agricultural Research Education and Extension Organization (AREEO), Karaj, Iran.

^c^ Department of Nanotechnology, Agricultural Biotechnology Research Institute of Iran (ABRII), Agricultural Research Education and Extension Organization (AREEO), Karaj, Iran.

*Corresponding authors:

Elaheh Motamedi ([motamedi.elaheh@gmail.com](mailto:motamedi.elaheh@gmail.com); e.motamedi@abrii.ac.ir)

Marzieh Ghollasi ([ghollasi@khu.ac.ir](mailto:ghollasi@khu.ac.ir))

*
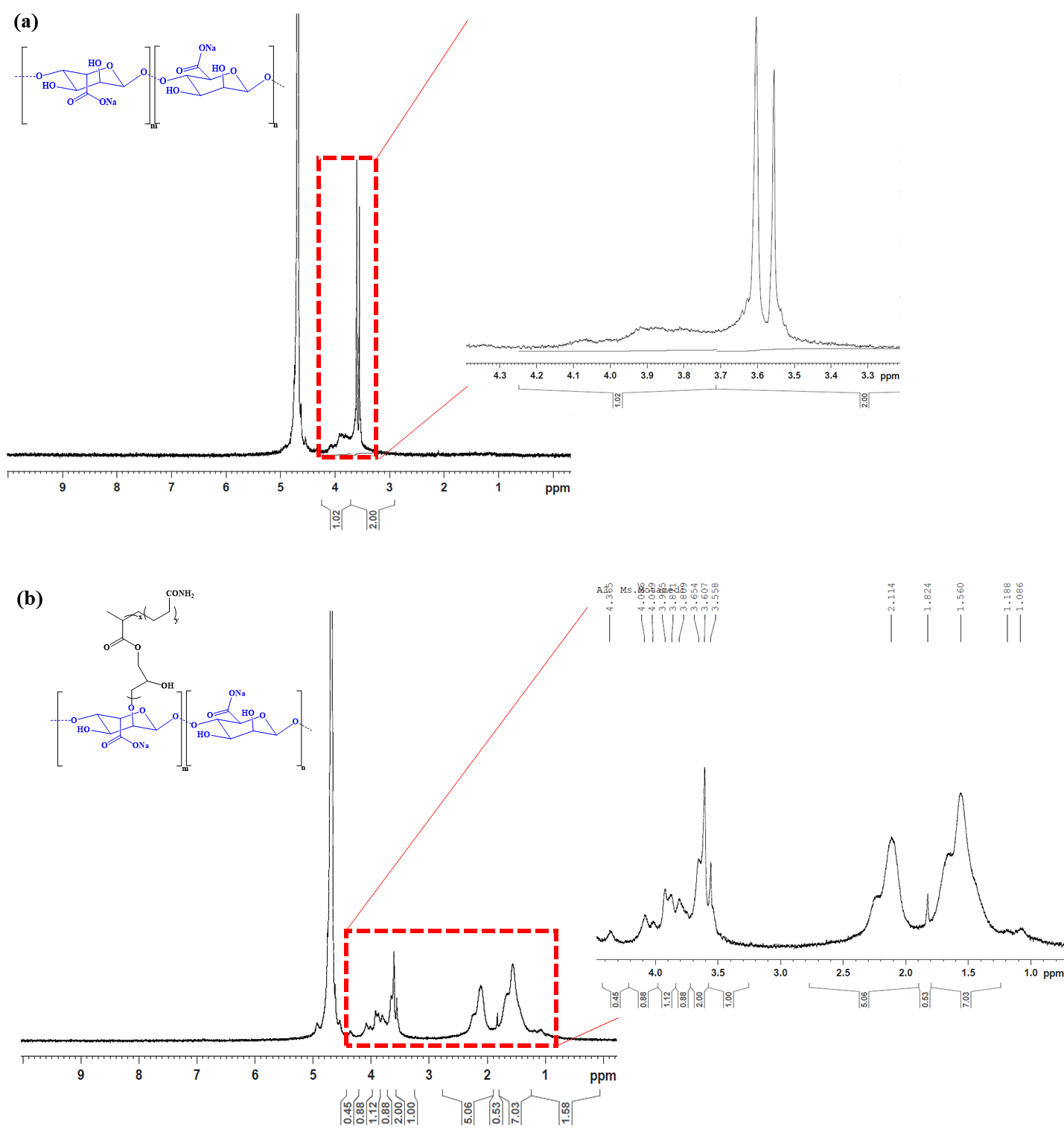
*

Fig. S1. 1H-NMR spectra of (a) sodium alginate and (b) SA-g-poly(AAm-co-GMA) hydrogel sample in D_2_O.

*
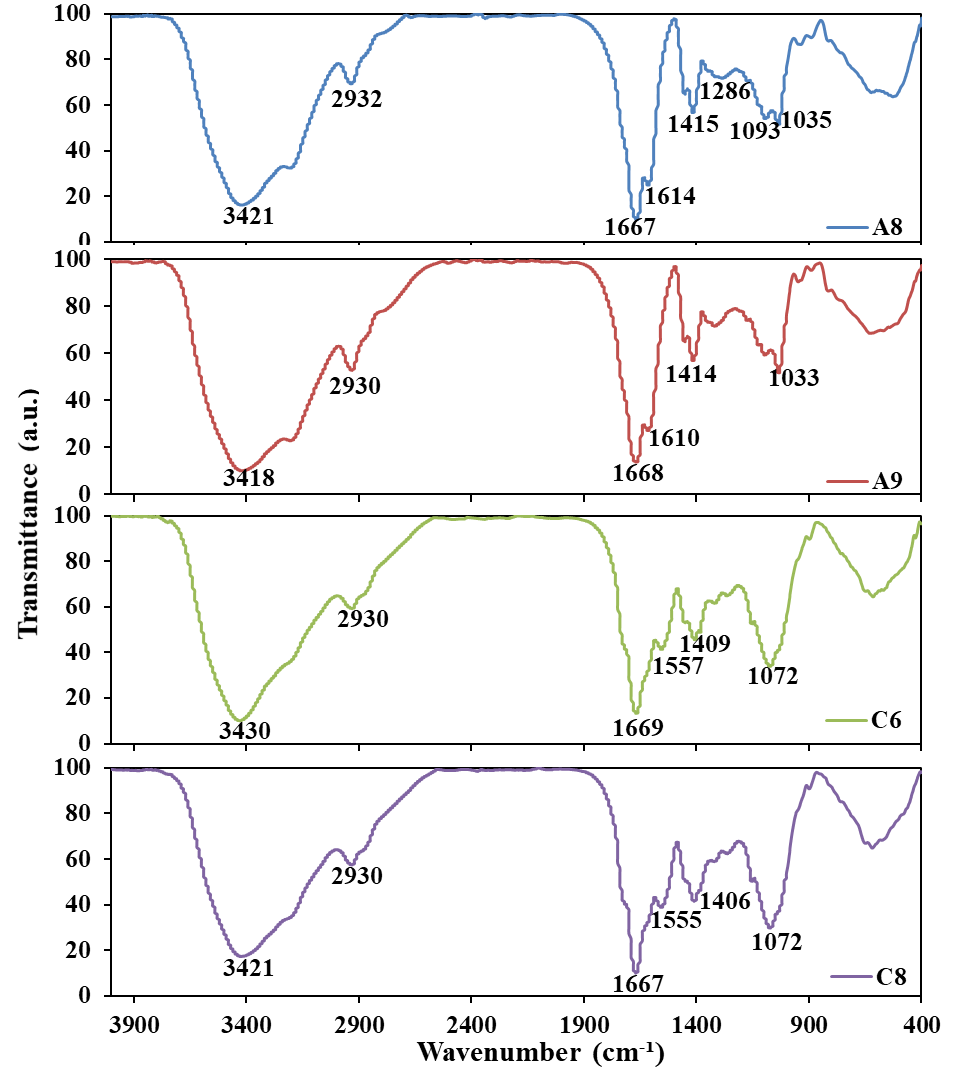
*

Fig. S2. FTIR spectra of selected SA-based hydrogels (A8, A9), and CH-based hydrogels (C6, C8).

*
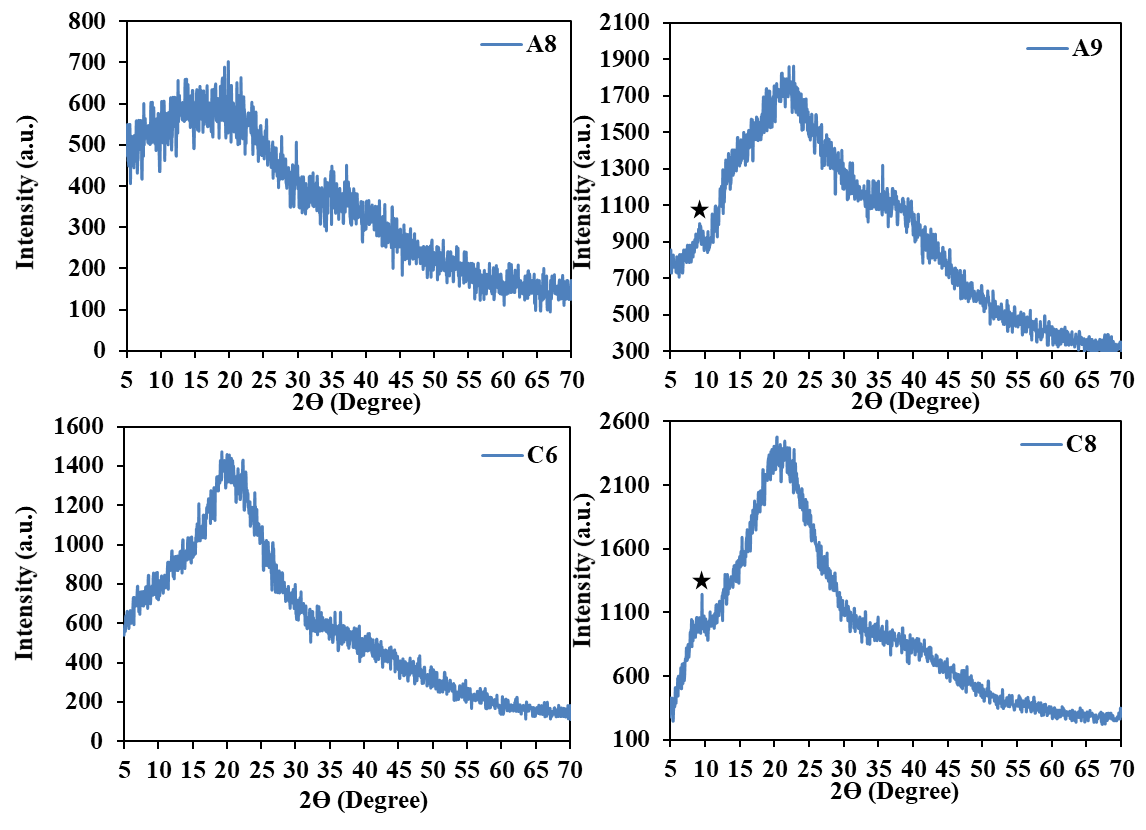
*

Fig. S3. XRD patterns of selected SA-based hydrogels (A8, A9), and CH-based hydrogels (C6, C8).
